# Contrastive pre-training and 3D convolution neural network for RNA and small molecule binding affinity prediction

**DOI:** 10.1093/bioinformatics/btae155

**Published:** 2024-03-20

**Authors:** Saisai Sun, Lin Gao

**Affiliations:** School of Computer Science and Technology, Xidian University, No.266 Xinglong Section of Xi Feng Road, Xi’an, Shaanxi, 710126, China; School of Computer Science and Technology, Xidian University, No.266 Xinglong Section of Xi Feng Road, Xi’an, Shaanxi, 710126, China

## Abstract

**Motivation:**

The diverse structures and functions inherent in RNAs present a wealth of potential drug targets. Some small molecules are anticipated to serve as leading compounds, providing guidance for the development of novel RNA-targeted therapeutics. Consequently, the determination of RNA–small molecule binding affinity is a critical undertaking in the landscape of RNA-targeted drug discovery and development. Nevertheless, to date, only one computational method for RNA–small molecule binding affinity prediction has been proposed. The prediction of RNA–small molecule binding affinity remains a significant challenge. The development of a computational model is deemed essential to effectively extract relevant features and predict RNA–small molecule binding affinity accurately.

**Results:**

In this study, we introduced RLaffinity, a novel deep learning model designed for the prediction of RNA–small molecule binding affinity based on 3D structures. RLaffinity integrated information from RNA pockets and small molecules, utilizing a 3D convolutional neural network (3D-CNN) coupled with a contrastive learning-based self-supervised pre-training model. To the best of our knowledge, RLaffinity was the first deep learning based method for the prediction of RNA–small molecule binding affinity. Our experimental results exhibited RLaffinity’s superior performance compared to baseline methods, revealed by all metrics. The efficacy of RLaffinity underscores the capability of 3D-CNN to accurately extract both global pocket information and local neighbor nucleotide information within RNAs. Notably, the integration of a self-supervised pre-training model significantly enhanced predictive performance. Ultimately, RLaffinity was also proved as a potential tool for RNA-targeted drugs virtual screening.

**Availability and implementation:**

https://github.com/SaisaiSun/RLaffinity

## 1 Introduction

RNA molecules, traditionally known for their roles in information transfer and protein synthesis, have emerged as key players in gene regulation, cellular signaling, and disease pathways. The functionality of RNA molecules is commonly realized through intricate interactions with various cellular components, encompassing proteins, peptides, DNAs, other RNAs, and small molecules. Notably, RNA–small molecule interactions play a crucial role in various biological processes and have garnered significant research interests in recent years (Morgan *et al.* 2017). Moreover, as our understanding of RNA structures and functions continues to accumulate, it becomes evident that RNAs are intricately linked to conditions such as cancer and viral diseases, functioning as mediators in neurological diseases, and emerging as potential targets for therapeutic interventions (Bernat and Disney 2015). The study of RNA–small molecule interactions can provide valuable insights into the mechanisms underlying RNA-mediated processes. On one hand, these interactions can occur through multiple modes, including hydrogen bonding, electrostatic interactions, hydrophobic interactions, and pi-stacking interactions (Szulc *et al.* 2022). On the other hand, small molecules can comprise a diverse range of compounds, including metabolites, drugs, and chemical probes. These molecules can bind to specific regions of RNA, such as hairpins, loops, or bulges, inducing conformational changes that can impact RNA folding, stability, and activity (Costales *et al.* 2020). They can also target functional RNA elements, such as ribozymes, riboswitches, or non-coding RNA domains, to regulate gene expression or modulate cellular processes. These multifaceted interactions allow for the design of small molecules with the capacity to selectively bind to RNA, enabling the inhibition or activation of specific RNA functions, offering a unique approach for precision medicine (Aboul-Ela 2010).

A plenty of studies have been conducted to identify RNA–small molecule interactions experimentally. An illustrative example is the work by Serganov *et al.*, where they presented the crystal structures of the Fusobacterium nucleatum riboswitch bound to riboflavin and the antibiotic roseoflavin (Serganov *et al.* 2009). And some studies have elucidated the roles of microRNA and long non-coding RNA in various human diseases (Tang *et al.* 2021, Gao *et al.* 2022, Fan *et al.* 2023). Simultaneously, some recent investigations have introduced small molecules and oligonucleotides into clinical trials for cancer treatment, with a focus on modulating the mRNA spliceosome (Lee *et al.* 2016, Effenberger *et al.* 2017). Subsequently, RNA has emerged as a spivotal category of potential therapeutic targets, as evidenced by comprehensive reviews (Thomas and Hergenrother 2008, Aboul-Ela 2010). Notably, to date, the landscape of FDA-approved drugs interacting with RNAs remains relatively limited, such as: risdiplam, patisiran, fomivirsen, inotersen, mipomersen, and eteplirsen (Zogg *et al.* 2022).

The analysis of RNA–ligand interactions is significantly enhanced by the existence of high-resolution structures of RNA–ligand complexes. Nevertheless, the experimental determination of structures for RNA and its complexes poses multiple challenges and currently cannot be achieved in a high-throughput manner. Consequently, the need to overcome these challenges has driven the development of computer software aimed at modeling RNA–ligand complex structures, based on the available structures of RNA receptors. Several of these advancements have been inspired by methods previously devised for modeling protein–ligand complexes (Bottegoni 2011). While recent years have witnessed significant strides in the development of automatic docking tools for predicting protein–ligand interactions (Gohlke and Klebe 2002, Sousa *et al.* 2006), the modeling of RNA–ligand interactions has seen comparatively less progress (Pfeffer and Gohlke 2007). Only a few appropriate docking and scoring methods for RNA–ligand interactions have been developed, such as rDOCK (Morley and Afshar 2004, Ruiz-Carmona *et al.* 2014), AutoDock (Trott and Olson 2010, Goodsell *et al.* 2021), MORDOR (Guilbert and James 2008), Dock6 (Lang *et al.* 2009), as well as NLDock (Feng *et al.* 2021). Correspondingly, the scoring functions for docking include LigandRNA (Philips *et al.* 2013), SPA-LN (Yan and Wang 2017), ITScore-NL (Feng and Huang 2020), and DrugScoreRNA (Pfeffer and Gohlke 2007). Additionally, a few databases focusing on RNA–ligand interactions have been established, such as SMMRNA (Mehta *et al.* 2014), HARIBOSS (Panei *et al.* 2022), RNALID (Fan *et al.* 2023), and RPocket (Zhou *et al.* 2021). Besides, several RNA–ligand binding sites prediction algorithms have been proposed, such as MetalionRNA (Philips *et al.* 2012), Rsite (Zeng *et al.* 2015), RBind (Wang *et al.* 2018), RNAsite (Su *et al.* 2021), RNALigand (Sun et al., 2022) and RLBind (Wang *et al.* 2023).

Despite the usefulness of these databases and methods, to date there has not been very active research in cataloging and predicting RNA and small molecule ligand interaction strength or affinity. To the best of our knowledge, only one computational approach predicting RNA–ligand binding strength or affinity has been developed. The method was named RSAPred, which was developed based on traditional machine learning (Krishnan *et al.* 2024). Comprehensive understanding of binding affinities to substrates, inhibitors, and cofactors is essential for unraveling the intermolecular interactions that drive biological processes, structural biology, and the intricate connections between structure and function in proteins, nucleic acids, and other biomolecules. However, several challenges exist in predicting RNA–small molecule binding affinities, such as the inherent flexibility and conformational dynamics of both RNA and small molecules impeding accurately capturing their interactions. Additionally, the limited availability of high-quality experimental data on RNA–small molecule binding affinities hinders the development of robust and generalizable prediction models.

In this study, we aim to address these challenges and contribute to the field of RNA–small molecule binding affinity prediction. We have developed a reliable and accurate prediction model named RLaffinity, by combing a contrastive learning-based self-supervised pre-training model and a 3D-CNN model. Firstly, our method was compared with some baseline methods and demonstrated superior performances. Additionally, we did a blind test on RNA–small molecule pairs, revealing consistent results with other literatures. Furthermore, our method was proved utility in drug discovery for ranking compound hits binding to the target, which demonstrated that RLaffinity can aid in the development of medicines that bind their targets selectively and precisely and enhance our understanding of RNA functions.

## 2 Materials and methods

### 2.1 RNA–ligand complexes

In our study, we employed the standardized data, specifically consisting of the structures of RNA and ligands in their bound state (i.e. complex state), archived in individual Protein Data Bank (.pdb) format files (Berman 2008, wwPDB consortium 2019). The strength of ligand binding to RNA was defined as binding affinity, which was determined for each complex structure through experimental techniques such as spectroscopic shift assays and isothermal titration calorimetry (Ballester and Mitchell). The experimental binding affinity data (dissociation constants $k_{d}$) were used as the ground truth labels for the supervised training and testing procedure. And following the works in protein–ligand binding affinity prediction (Jiménez *et al.* 2018, Jones *et al.* 2021), the dissociation constants $k_{d}\;$were normalized by the negative log function [i.e. $-\log(k_{d})$]. In addition, for the effectiveness input of the neural networks, all labels were normalized to (0, 1) using the min-max normalization method.

In the initial phase, RNA–small molecule pairs lacking binding affinity data were downloaded and filtered from Protein Data Bank (PDB) for the self-supervised pre-training process. This phase involved exclusive training with RNA–ligand structures, devoid of affinity labels. Firstly, all structures containing both RNA and small molecules were downloaded from PDB, obtaining 1429 RNA–small molecule complex structures. Subsequently, structures containing proteins or RNAs with lengths less than 10 nucleotides were selectively removed. Following this refinement, all retained structures exclusively featured ions as their ligands, and any structures containing only artifact ligands were excluded. Ultimately, 1415 complex structures with interatomic distances between RNA and ligand smaller than 4 Å were preserved for subsequent pre-training procedure.

In the second phase, RNA–small molecule pairs with binding affinity labels were obtained from the PDBbind database for the supervised training and testing processes (Wang *et al.* 2004, 2005). Specifically, 149 RNA–ligand pairs were retrieved from PDBbind NL2020 set, including 144 compounds, one DNA and four peptides as the ligands. The distribution of these RNA lengths was shown in Supplementary Fig. S3. Subsequently, 144 RNA–ligand pairs were remained after removing the DNA ligand and peptide ligands. After that, 100, 29, and 13 pairs were randomly selected for training, validation, and testing, respectively. Given the limited dataset, this procedure was iterated 10 times to generate 10 distinct training sets, 10 distinct validation sets, and 10 corresponding test sets. During the training procedure, the optimal parameters were obtained according to the performances of the validation sets. And then the final 10 models with the optimal parameters were tested on the 10 corresponding test sets. Notably, no redundancy elimination procedure was applied, considering the absence of substantial similarity between RNA–ligand pairs. Additionally, to augment the original data, rotational and translational transformations were incorporated into the training procedure to expand the labeled dataset.

### 2.2 Binding pocket, features, box size, and grid resolution

To obtain accurate three-dimensional representations for the deep learning models, a standardized preprocessing procedure was implemented on the binding complex structures deposited in Protein Data Bank (.pdb) format.

Firstly, all RNA–ligand binding complexes were charged and protonated by UCSF Chimera (Couch *et al.* 2006) with AMBER bsc1 (Oweida *et al.* 2021), with the default settings of the program. Following this, for each RNA–ligand complex, KD-Trees algorithm was utilized to detect of the binding pocket, with a designated query radius of 10 Å (distance from the ligand atoms). The KD-Tree algorithm is a data structure designed for organizing points in *k*-dimensional space in a hierarchical manner. This structure greatly simplifies and enhances efficiency in tasks such as closest-neighbor searches, which involve identifying the nearest neighbors of a given point within the space (Bentley 1975).

Then, a standardized atomic representation was employed as input for the 3D-structure-based deep learning models. Specifically, only the heavy atoms from each biological structure were taken into consideration. This involved employing a one-hot encoding scheme for eight major elements, including C, N, O, P, F, Cl, Br, and I. The cheminformatic tool OpenBabel (version 2.4.1) (O’Boyle *et al.* 2008) was employed to extract features for all binding complexes. Subsequently, to generate the spatial representation of the binding complex, all atomic coordinates were centered by each ligand.

Furthermore, to determine the voxel box size, the distribution of end-to-end distances for all ligands in our datasets were calculated, as shown in Supplementary Figs S1 and S2. According to the sum of the end-to-end distance for the longest ligand in these two datasets and the query radius, the voxel box size was ultimately defined as 41 Å, which is sufficient to cover the entire pocket region while minimizing the collisions between atoms. If the dimensions of the box be set smaller than the length of a ligand, there exists the possibility that the terminal sides of the ligand to extend beyond the space of the box, resulting in data loss about molecular components. Even in cases where the initial orientation permits the ligand to fit within the box, subsequent data augmentation procedures are likely to generate input structures with consequential data loss.

The Van der Waals radius of the eight major heavy atoms (C, N, O, P, F, Cl, Br, and I) used in our study are greater than 1.4 Å. This radius is considered a measure of an atom’s size, defined as half of the internuclear separation of two non-bonded atoms of the same element at their closest possible approach. A grid resolution larger than two times of the radius would be insufficient to differentiate two atoms from each other. Conversely, opting for a finer resolution would result in a significantly higher computational cost. To ensure an optimal balance between accuracy and efficiency, we set the 3D voxel grid resolution at 1.0 Å. Hence, each atom can be allocated to at least one voxel grid, depending on its Van der Waals radius. Subsequently, after the voxelization of all atoms, a Gaussian blur with σ=1 was employed to diffuse the atom features into neighboring voxels. This strategy was implemented to prevent an excessively sparse representation within the 3D voxel grid, similar to that employed in the previous work (Kuzminykh *et al.* 2018).

### 2.3 Model architecture

**Figure 1.**
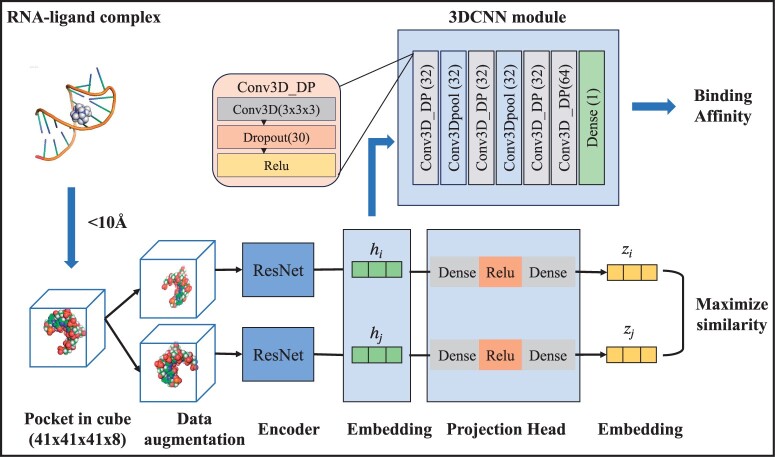
The flowchart of RLaffinity. Firstly, RNA-ligand complex structures without labels were represented as cubes with a dimension of 41 × 41 × 41 × 8 and input into a self-supervised pre-training model, here referring to a contrastive learning model, for RNA–ligand interaction information extraction. After the pre-training model, RNA–ligand complex structures with labels were input into the pre-trained model to generate structural representations ($h_{i}$), which then were input to the 3DCNN-based regression models for binding affinity prediction.

To sufficiently utilize the unlabeled RNA–ligand structures data, an unsupervised pre-training model was designed for the RNA–ligand interaction information and pattern extraction. Subsequently, a supervised regression model was employed and fine-tuned to predict RNA–ligand binding affinities. For the unsupervised pre-training, a contrastive learning model was utilized in this study, demonstrating its ability to generate efficient 3D representations for the subsequent supervised regression model. Furthermore, the prediction of binding affinity was performed through a three-dimensional convolutional neural network model. An illustration of the overall model architecture is presented in Fig. 1.

### 2.4 Three-dimensional convolutional neural networks

3D-CNNs have been extensively applied to diverse computer vision fields, such as gestures or shapes recognition in videos and 3D image segmentation, due to its capability of shift invariance and representation learning. Lately, 3D-CNNs have been substantiated for their efficacy in predicting binding affinity and elucidating protein–ligand interactions within the domain of drug discovery (Jiménez *et al.* 2017, 2018, Ragoza *et al.* 2017, Kuzminykh *et al.* 2018, Jones *et al.* 2021). The inherent capability of the 3D-CNN model lies in its ability to capture intricate three-dimensional atomic features and implicit atomic interactions. This is achieved through the use of 3D volume representations, wherein atoms and their respective features are voxelized into a three-dimensional voxel grid. The 3D atomic representation dimension was *N* × *N* × *N* × *C*, where *N* denoted the voxel box size in each axis, and *C* represented the number of atomic features described in the preceding section (eight in our study).

To build the supervised regression model, a straightforward 3D-CNN architecture was implemented. This model consisted of only four convolutional layers, complemented by four dropout layers to avoid overfitting, as visually represented in Fig. 1(top). To enhance the model’s nonlinear capacity, a rectified linear unit (RELU) layer was integrated within each convolutional and fully connected layer. Additionally, to ensure translation invariance, MaxPooling layer was applied to the second and fourth convolutional layers. The adaptive moment estimation (Adam) optimizer, with a learning rate set to 0.0001, was employed for optimization. The batch size, denoting the number of samples processed per batch, was configured as 16. The overall parameters of the 3D-CNN model can be found in Supplementary Table S1, including details such as filter size, kernel size and stride.

### 2.5 Contrastive learning framework

Incorporating insights from recent advances in computer vision (Wang *et al.* 2021, Xie *et al.* 2021, Zhao *et al.* 2021, Dave *et al.* 2022, Denize *et al.* 2023), our framework was trained with the objective of maximizing the similarity between representations derived from augmented structures of the same pocket–ligand complex while minimizing the similarity between augmented structures from different pocket–ligand complexes, referring to Fig. 1(bottom). Generally, for a given pocket–ligand complex cube, we performed rotations and translations of the complex within the cube to obtain two augmented structures, $S_{i}$ and $S_{j}$. Subsequently, we computed the latent representations of these augmented structures, $h_{i}$ and $h_{j}$, utilizing a 3D ResNet-based encoder, $h_{i}=R(S_{i})$. Following the previous approach in protein representation (Xia *et al.* 2022), these latent representations were further projected into smaller latent representations, $z_{i}$ and $z_{j}$, using a multilayer perceptron with a single hidden layer, $z_{i}=P(h_{i})$. Ultimately, the similarity between these representations was quantified using the cosine distance, denoted as $s(z_{i},z_{j})$ and the associated training loss was determined through the computation of the NT-Xent loss (Denize *et al.* 2023). The detailed parameters involving in the contrastive learning model can be found in Supplementary Table S2.

Specifically, the 3D residual neural network (ResNet18) model served as the encoder within the contrastive learning framework, comprising a total of eight residual blocks (Alaeddine and Jihene 2021). The inclusion of the residual short connection facilitates the transmission of gradients to subsequent layers without engaging nonlinear activation. Each residual block was structured with three convolution layers, three batch normalization layers, and three RELU activation layers. The final output dimension of this module was 23 × 23 × 23 × 32.

### 2.6 Evaluation metrics

In this work, to evaluate the performance of our method and compare it with other baseline methods, we adopted the widely used evaluation metrics in the protein-ligand binding affinity prediction task, including Pearson correlation coefficient (PCC), Spearman correlation coefficient (SPCC), root mean square error (RMSE), and absolute error (AE). The definitions of these four metrics are defined as follows:

$$\begin{array}{l} P\mathrm{CC}=\frac{\sum_{i=1}^{n}\left({\mathrm{true}}_{i}-\bar{\mathrm{true}}\right)\left({\mathrm{predict}}_{i}-\bar{\mathrm{predict}}\right)}{\sqrt{\sum_{i=1}^{n}{\left({\mathrm{true}}_{i}-\bar{\mathrm{true}}\right)}^{2}}\sqrt{\sum_{i=1}^{n}{\left({\mathrm{predict}}_{i}-\bar{\mathrm{predict}}\right)}^{2}}}\\ \begin{array}{l} \mathrm{SPCC}=\frac{\sum_{i=1}^{n}\left(R_{i}-\bar{R}\right)\left(S_{i}-\bar{S}\right)}{\sqrt{\sum_{i=1}^{n}\left(R_{i}-\bar{R}\right)}\sqrt{\sum_{i=1}^{n}\left(S_{i}-\bar{S}\right)}}\\ \begin{array}{l} \mathrm{RMSE}=\sqrt{\frac{1}{n}\sum_{i=1}^{n}{\left({\mathrm{true}}_{i}-{\mathrm{predict}}_{i}\right)}^{2}}\\ AE=\left|{\mathrm{true}}_{i}-{\mathrm{predict}}_{i}\right| \end{array} \end{array} \end{array}$$

where n is the number of RNA–ligand samples in the dataset, ${\mathrm{true}}_{i}$ refers to the experimentally measured binding affinity of the sample indexed with i, and ${\mathrm{predict}}_{i}$ refers to the predicted binding affinity of the sample indexed with i, $\bar{\mathrm{true}}$ refers to the mean of the experimentally measured binding affinity of n samples, and $\bar{\mathrm{predict}}$ refers to the mean of the predicted binding affinity of n samples, $R_{i}$ refers to the rank of the experimentally measured binding affinity of the sample indexed with i, $S_{i}$ refers to the rank of the predicted binding affinity of the sample indexed with i, $\bar{R}$ refers to the mean rank of the experimentally measured binding affinity of n samples, $\bar{S}$ refers to the mean rank of the predicted binding affinity of n samples.

## 3 Results and discussions

### 3.1 Comparison with other baseline methods

To evaluate the robustness of our method, we trained 10 models and conducted testing on 10 distinct sets randomly sampled from the entire dataset. Table 1 presents the mean metrics values across the 10 test sets for different methods, including Vina (Trott and Olson 2010), RF-score (Ballester and Mitchell 2010), RSAPred (Krishnan *et al.* 2024), our 3D-CNN model, and our pre-trained 3D-CNN model (RLaffinity). Evidently, the 3D-CNN models, both in its conventional form and when augmented with pre-training, exhibit heightened predictive capabilities (PCCs: 0.466 and 0.559) compared to Vina, RF-score, and RSAPred (PCCs: −0.386, 0.445, and 0.399). The increased correlation coefficients (PCCs/SPCCs) and reduced RMSE values associated with 3D-CNN indicate its efficacy in providing accurate predictions for binding affinities of RNA and small molecules. Furthermore, the self-supervised pre-training before the 3D-CNN model demonstrates potential enhancements, as indicated by the increased correlation coefficients (PCCs/SPCCs). This underscores the viability of leveraging pre-existing structural information or representations to enhance predictive performance.

**Table 1.** The results from different methods on the benchmark test set.^a^

Methods	PCC	SPCC	RMSE	MAE
Vina	−0.386	−0.389	0.277	0.257
RF-score	0.445	0.364	0.152	0.129
RSAPred	0.399	0.221	2.886	2.205
3DCNN	0.466	0.408	0.165	0.129
RLaffinity	**0.559**	**0.540**	**0.152**	**0.119**

a The value of each metric is the mean value of metrics on the 10 test sets. For Vina, we took the docking score as the predicted affinity. And the method of 3DCNN represents only using the 3DCNN model for prediction. For the RSAPred method, the labels were not normalized leading to RMSE and MAE values exceeding 1. The highest value of each metric from different methods is highlighted in bold type.

**Figure 2.**
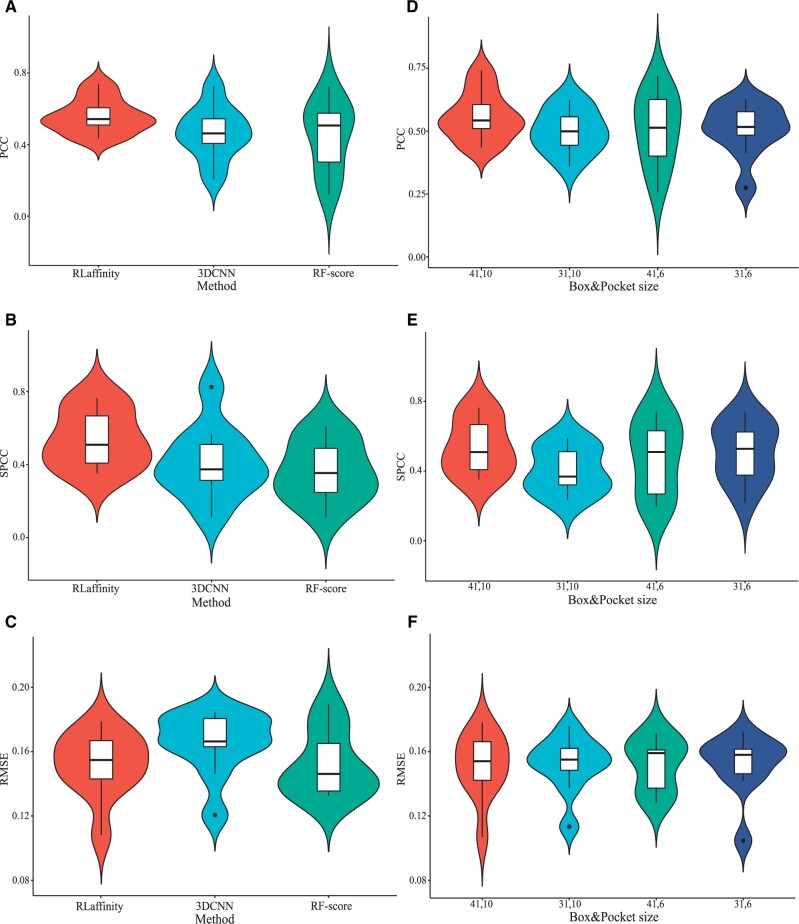
The metrics distributions of different methods (A–C) and different parameters (D–F). Here, RLaffinity, 3DCNN and RF-score represent our method with pre-training, our method without pre-training and the method based on random forest, respectively. And the numbers in horizontal axis of figure (D–F) represent different combinations of box size and pocket size.

To further explore the details of the results, we analyzed the metric distributions across different methods and parameters on 10 test sets. Figure 2A–C present violin plots illustrating the distributions, scopes, and median values of PCC, SPCC, and RMSE for various methods. Notably, our pre-trained 3D-CNN model (RLaffinity) demonstrates the narrowest deviation scope for each metric and the highest median values of correlation coefficients (PCC: 0.540, SPCC: 0.500), compared to 3D-CNN model (PCC: 0.460, SPCC: 0.360), and RF-score (PCC: 0.480, SPCC: 0.320). This indicates that our pre-training model provided more stable and accurate predictions of RNA–ligand binding affinity. Additionally, the 3D-CNN method exhibits a more robust result in PCC distribution and slightly higher SPCC values compared to the RF-score method, which are consistent with the results showed in Table 1. As for the RMSE distributions, RF-score method has a higher maximum value than the other two methods. And most RMSE values focus on similar ranges for RLaffinity method and RF-score method (0.120–0.180), with 0.155/0.144 as the medium value. In summary, these observations suggest that 3D convolutional neural networks can effectively extract valuable information about binding strength from complex structures for the prediction of binding affinities between RNA and small molecules.

**Figure 3.**
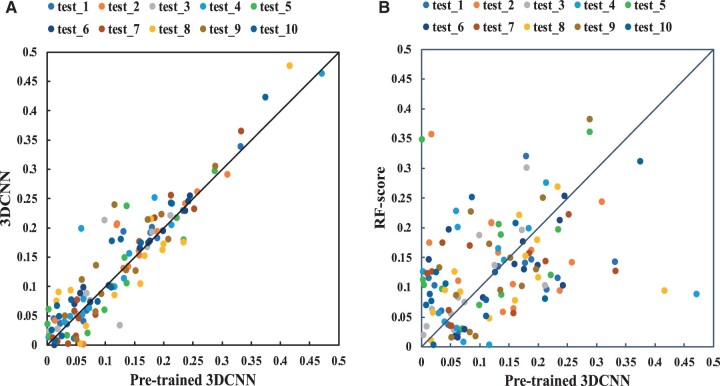
One-to-one AE comparison of different methods on 10 test sets. Here, the horizontal axis represents pre-trained 3DCNN method (RLaffinity) and the vertical axis represents 3DCNN method without pre-training (A) and the RF-score method (B).

Furthermore, for a more direct comparison of methods, we did a one-to-one comparison of the pre-trained 3D-CNN method with the original 3D-CNN method and the RF-score method. Figure 3 presents the scatter plots of the AE values of different methods on 10 test sets. In Fig. 3A, the majority of dots concentrate in the upper region, indicating that most samples exhibit higher AE values generated by 3D-CNN method compared to the pre-trained 3D-CNN method. This highlights the substantial improvement in performance achieved through the pre-training procedure. However, Fig. 3B indicates a comparable number of dots falling in both the upper and lower regions, including some notable dots with large AE values. For these specific samples, further analysis and improvement are warranted.

### 3.2 Pocket size and box size selection

In the investigation of protein–small molecule interactions, a conventional radius of 6 Å has been widely employed to extract binding pockets. In this study, we conducted a systematic examination of different pocket sizes (6 and 10 Å) to identify the most suitable parameter for predicting RNA–small molecule binding affinity. Furthermore, considering that 3D-CNNs process four-dimensional input data (*N* × *N* × *N* × *C*, where *N* denotes the box size and *C* represents the channels), variations in box sizes can impact feature extraction through 3D-CNN. In consideration of the ligand lengths distribution, we opted for box sizes of 31 and 41 Å, chosen to encompass the entirety of both pocket and ligand structures. The comparative results based on diverse pocket sizes and box sizes are presented in Table 2. Notably, the highest PCC of 0.559 was observed when using a pocket size of 10 Å and a box size of 41 Å. Additionally, distinct parameters yielded similar RMSEs around 0.15.

**Table 2.** The results from different parameters on the benchmark test set.^a^

Pocket size Box size	6 Å	10 Å
**31 Å**	0.507 (0.153)	0.500 (0.153)
**41 Å**	0.510 (0.153)	**0.559** (0.152)

a The value of each metric is the mean value of metrics on the 10 test sets.  The highest value of each metric from different parameters is highlighted in bold type.

For a more comprehensive understanding of the results obtained with different box sizes and pocket sizes, we delved into the metrics distributions across all test sets with various parameter combinations. The outcomes are visually depicted in Fig. 2D–F through violin plots. In Fig. 2, a pocket size of 10 Å exhibits a narrower range across both correlation coefficients (PCCs and SPCCs) compared to a pocket size of 6 Å. This observation suggests that a pocket size of 6 Å might inadvertently exclude some crucial atoms involved in essential chemical bonds, potentially leading to less stable predictions. Regarding box sizes, a size of 41 Å, in contrast to a size of 31 Å, achieved a higher median value of both PCCs (0.534, 0.483) and SPCCs (0.500, 0.360) with the pocket size of 10 Å. These results suggest that larger box sizes (41 Å) coupled with smaller pocket size (6 Å) resulted in broader ranges of PCCs/SPCCs might be due to sparser embeddings within the grid box. Conversely, box size of 31 Å coupled with pocket size of 10 Å may result in information omitting. In addition, all distinct size parameters demonstrated similar performances in RMSE distributions, with a range around 0.08–0.20 and a medium value of 0.154.

**Figure 4.**
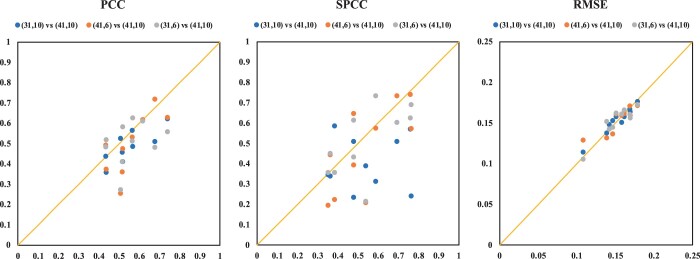
Metrics comparisons (PCC, SPCC, and RMSE) of different combinations of box size and pocket size on 10 test sets.

To further assess the impact of different box and pocket size combinations, we conducted one-to-one comparisons on all metrics across various test sets. Figure 4 illustrates scatter diagrams depicting PCCs, SPCCs, and RMSEs for different box size and pocket size combinations. From Fig. 4A and B, it is evident that the combination of a box size of 41 Å and a pocket size of 10 Å outperforms other combinations in terms of PCC and SPCC metrics. This suggests that a box size of 41 Å and a pocket size of 10 Å exhibit superior performance across most test sets. Additionally, Fig. 4C indicates that the combination of a box size of 41 Å and a pocket size of 10 Å demonstrates similar RMSE values when compared to other combinations on each test set.

### 3.3 Blind test on structure unknown complexes

**Figure 5.**
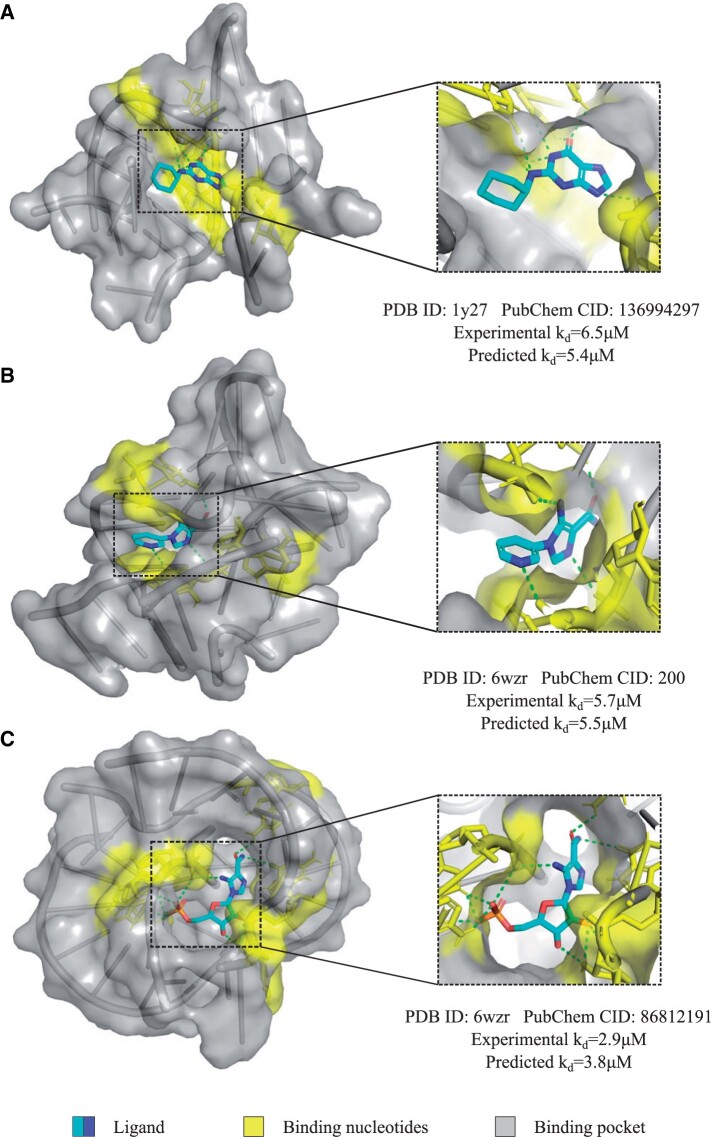
Blind tests on three RNA–small molecule pairs. Thereinto, the 3D interaction structures were generated by Vina. (A) is a “G-riboswitch-guanine analogue” complex, (B) and (C) are two “Fusibacterium ulcerans ZTP riboswitch-5-aminoimidazole-4-carboxamide (AICA) analog” complexes.

To further validate the effectiveness of our method, we conducted a blind test on three RNA–small molecule pairs from literatures (Yan *et al.* 2018, Tran *et al.* 2020). Firstly, the RNA and small molecule structures were docked by Autodock Vina to generate the complex structures with default parameters (Trott and Olson 2010). Subsequently, leveraging the known structures as input, we predicted the binding pockets and binding affinities by our method. Figure 5 exhibits the docking results of the three RNA–ligand pairs, which include a “G-riboswitch-guanine analog” complex (Fig. 5A) and two “Fusibacterium ulcerans ZTP riboswitch-5-aminoimidazole-4-carboxamide (AICA) analog” complexes (Fig. 5B and C). They are reported involving in bacterial growth and infection related diseases (Tran *et al.* 2020). The guanine derivatives were deciphered structure–activity relationship with the guanine riboswitches and inhibitory effect on bacterial growth through in-line probing experiments (Tran *et al.* 2020). And the AICA derivatives were found to bind and activate ZTP riboswitches *in vitro* through transcription termination assays (Yan *et al.* 2018). As depicted in Fig. 5, the “G-riboswitch-guanine analog” complex, with an assay-measured $k_{d}$ of 6.5 µM, exhibited a medium-strength affinity. Our method predicted its binding affinity with a $k_{d}$ of 5.5 µM, indicating a slight deviation. Specifically, the head of its ligand was positioned outside its pocket, resulting in limited interactions between the tail of the ligand and the receptor. In the case of the “Fusibacterium ulcerans ZTP riboswitch-5-aminoimidazole-4-carboxamide (AICA) analog” complexes, their binding affinities were measured by assay with $k_{d}$ ∼5.7 and ∼2.9 µM. And their prediction values were 5.4 and 3.8 µM, respectively, demonstrating the capability of our approach to provide effective information for RNA drug selections. To be more specific, the AICA analog ligands were entirely enveloped by their pockets, facilitating well-proportioned and high-density interactions between the ligands and their respective binding sites. Moreover, AICA analog five, in comparison to AICA analog 13, features a greater number of hydroxide radicals. This characteristic contributes to the formation of more hydrogen bonds with the receptor, resulting in a slightly higher binding strength. Altogether, these findings could serve as guidance for the future design of enhanced riboswitch activators and offer valuable insights into the potential trajectory of RNA-targeted ligand discovery.

### 3.4 Potential compounds screening

**Figure 6.**
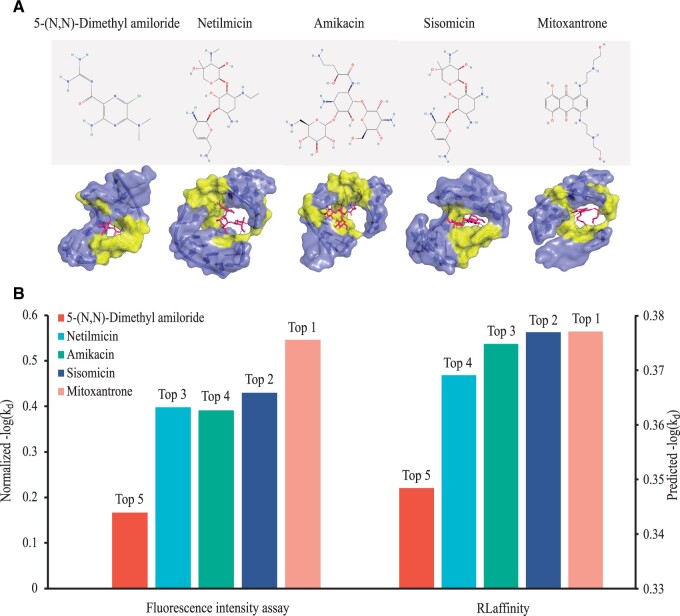
Compounds selection against HIV-1 TAR target. (A) 2D structures of the five compound hits targeting HIV-1 TAR and their 3D interaction structures visualized by Pymol (slate: pocket, yellow: interaction sites, magenta: ligand, black: hydrogen bonds). (B) Ranking results of the five compounds from the experimental assay (left) and from our method (right).

Furthermore, we conducted a virtual screening of small molecules targeting the entire structural landscape of the transactivation response element (TAR) from the human immunodeficiency type 1 virus (HIV-1) (Stelzer *et al.* 2011). We performed quantitative predictions of binding affinities for small molecules that interact with HIV-1 TAR and reported the top five ranking compounds that bind TAR with near record affinities. Figure 6 provides comprehensive binding information of potential compounds [5-(*N*, *N*)-dimethyl amiloride, netilmicin, amikacin, sisomicin, mitoxantrone] with the target (HIV-1 TAR). In Fig. 6A, 2D chemical structures of the five small molecule hits are depicted along with the 3D interaction structures of each compound and its corresponding pocket within the HIV-1 TAR (generated from Vina). Figure 6A indicates that each RNA–ligand complex formed a distinct binding pocket wrapping the corresponding compound with a specific binding mode.

Additionally, Fig. 6B exhibits the binding affinities ($-\log(k_{d})$) of the five compounds and their order ranked by the fluorescence intensity assay and RLaffinity. Among the tested compounds, 5-(*N*, *N*)-dimethyl amiloride displayed the highest $k_{d}$ value (121.85 µM), indicating the weakest binding strength with HIV-1 TAR. Netilmicin, Amikacin, and Sisomicin showed similar $k_{d}$ values at a moderate level (1.35, 1.54, and 0.73 µM). Mitoxantrone exhibited the lowest $k_{d}$ value of 0.076 µM, designating it as the top-ranked compound for targeting HIV-1 TAR RNA. Importantly, our method yielded a comparable ranking order for the five compounds based on the predicted binding affinities. This outcome underscores the utility of our method in facilitating virtual screening efforts effectively.

## 4 Conclusions

In conclusion, our comparative analysis provided significant insights into the strengths and limitations of diverse prediction methods of RNA and small molecule binding affinities. These findings could serve as a valuable contribution to the continual refinement of computational approaches in the domain of molecular binding predictions. It is crucial to contextualize these observations within the specific attributes and requirements of the investigated RNA–small molecule interactions. Subsequent research endeavors and validation studies are warranted to affirm these conclusions and potentially enhance the predictive models.

Notably, this study pioneers a 3D convolutional neural network (3D-CNN) model coupled with a contrastive learning-based self-supervised pre-training model for the precise prediction of RNA–small molecule binding affinities, presented in the RLaffinity framework. Leveraging structural information from both RNA pockets and small molecules, RLaffinity outperforms established baseline methods in the realm of binding affinity prediction. Combing with the appropriate box size and pocket size, the 3D-CNN model could extract global pocket information and local neighbor nucleotide information within RNAs. Additionally, the incorporation of a self-supervised pre-training model emerges as a key contributor to the heightened predictive performance, underscoring the efficacy of this approach. The versatility of RLaffinity is further highlighted by its potential application as a tool for virtual screening of RNA-targeted drugs. This research represents a significant stride forward in the computational prediction of RNA–small molecule interactions, offering valuable insights and paving the way for enhanced approaches to RNA-targeted drug discovery and development.

Key Points

- We proposed the first computational method named RLaffinity to predict the RNA–small molecule binding affinity.
- RLaffinity was constructed through a fusion model, including a contrastive learning-based pre-training model and a 3D-CNN regression model.
- Experimental results demonstrated that RLaffinity significantly outperformed other baseline methods and showed its potential use in RNA-targeted drug virtual screening.

## Supplementary Material

btae155_Supplementary_Data

## Data Availability

The codes and datasets are available online at https://github.com/SaisaiSun/RLaffinity.
