# Supplementary material for: Contrastive pre-training and 3D convolution neural network for RNA and small molecule binding affinity prediction
Source: Bioinformatics. 2024 Mar 20;40(4):btae155. doi: 10.1093/bioinformatics/btae155 (PMC11007238; doi:10.1093/bioinformatics/btae155)
Supplement: btae155_Supplementary_Data [file btae155_supplementary_data.docx]

**Supplementary Data**

Table S1. 3D-CNN model parameters.

| Parameters | Optimal value |
| --- | --- |
| Learning rate | 1e-4 |
| Dropout rate | 0.1 |
| Batch size | 16 |
| Filter size | 32, 32, 64,128 |
| Kernel size | (3,3,3) |
| Stride | (1,1,1) |

Table S2. Contrastive learning model parameters.

| Parameters | Optimal value |
| --- | --- |
| Batch size | 100 |
| Learning rate | 1e-3 |
| Weight decay | 1e-6 |
| Max epoch | 20 |

Figure S1. Ligand length distribution on dataset from PDB.

Figure S2. Ligand length distribution on dataset from PDBbind.

Figure S3. RNA length distribution on dataset from PDBbind.
